# 96101 Temporal Evolution of Neural Activity in Human Brain Organoids

**DOI:** 10.1017/cts.2021.464

**Published:** 2021-03-30

**Authors:** Kobina G. Mensah-Brown, James Lim, Dennis Jgamadze, Guo-li Ming, Hongjun Song, John A. Wolf, Han-Chiao I. Chen

**Affiliations:** University of Pennsylvania

## Abstract

ABSTRACT IMPACT: This study will provide the essential characterization of intrinsic neural activity in human brain organoids, both at the single cell and network levels, to harness for translational purposes. OBJECTIVES/GOALS: Brain organoids are 3D, stem cell-derived neural tissues that recapitulate neurodevelopment. However, to levy their full translational potential, a deeper understanding of their intrinsic neural activity is essential. Here, we present our preliminary analysis of maturing neural activity in human forebrain organoids. METHODS/STUDY POPULATION: Forebrain organoids were generated from human iPSC lines derived from healthy volunteers. Linear microelectrode probes were employed to record spontaneous electrical activity from day 77, 100, and 130 organoids. Single unit recordings were collected during hour-long recordings, involving baseline recordings followed by glutamatergic blockade. Subsequently, tetrodotoxin, was used to abolish action potential firing. Single units were identified via spike sorting, and the spatiotemporal evolution of baseline neural properties and network dynamics was characterized. RESULTS/ANTICIPATED RESULTS: Nine organoids were recorded successfully (n=3 per timepoint). A significant difference in number of units was seen across age groups (F (2,6) = 6.4178, p = 0.0323). Post hoc comparisons by the Tukey HSD test showed significantly more units in day 130 (51.67 ±14.15) than day 77 (16.33 ±14.98) organoids. Mean firing rates were significantly different in organoids based on age, with drug condition also trending toward significance (F (6,12) = 9.97; p = 0.0028 and p = 0.08 respectively). Post hoc comparisons showed a higher baseline firing rate in day 130 (0.99Hz ±0.30) organoids than their day 77 counterparts at baseline (0.31Hz ±0.066) and glutamate blockade (0.31Hz ±0.045). Preliminary network analysis showed no modularity or small-world features; however, these features are expected to emerge as organoids mature. DISCUSSION/SIGNIFICANCE OF FINDINGS: Initial analysis of brain organoid activity demonstrates changes in single unit properties as they mature. Additional work in this area, as well as further network analyses, will confer better sense of how to rationally utilize brain organoids for translational purposes.

